# Carbon Nanotubes as a Solid-State Electron Mediator for Visible-Light-Driven Z-Scheme Overall Water Splitting

**DOI:** 10.1021/jacs.4c03437

**Published:** 2024-05-15

**Authors:** Lihua Lin, Yiwen Ma, Nobuyuki Zettsu, Junie Jhon M. Vequizo, Chen Gu, Akira Yamakata, Takashi Hisatomi, Tsuyoshi Takata, Kazunari Domen

**Affiliations:** † Research Initiative for Supra-Materials, Interdisciplinary Cluster for Cutting Edge Research, Shinshu University, Nagano 380-8553, Japan; ‡ Department of Materials Chemistry, Faculty of Engineering, Shinshu University, Nagano 380-8553, Japan; § Energy Land-scape Architectonics Brain Bank, Shinshu University, Nagano 380-8553, Japan; ∥ Faculty of Natural Science and Technology, Okayama University, Kita-ku, Okayama 700-8530, Japan; ⊥ Office of University Professors, The University of Tokyo, Tokyo 113-8656, Japan

## Abstract

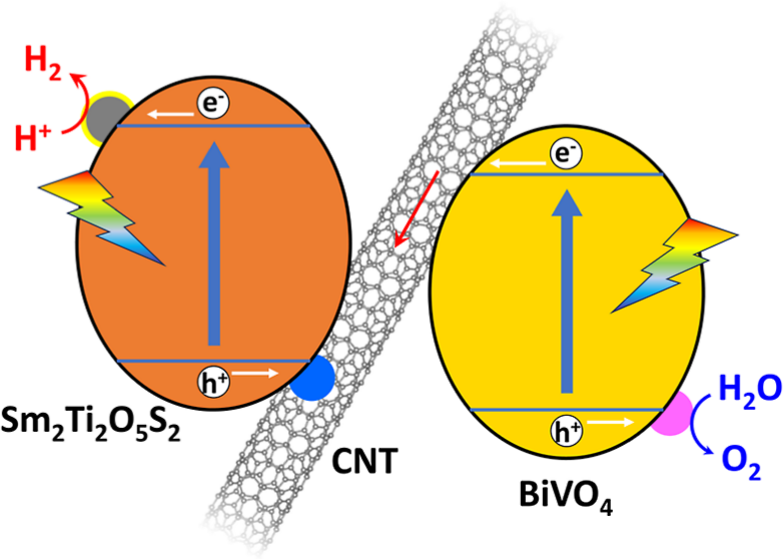

So-called Z-scheme systems, which typically comprise an H_2_ evolution photocatalyst (HEP), an O_2_ evolution photocatalyst (OEP), and an electron mediator, represent a promising approach to solar hydrogen production via photocatalytic overall water splitting (OWS). The electron mediator transferring photogenerated charges between the HEP and OEP governs the performance of such systems. However, existing electron mediators suffer from low stability, corrosiveness to the photocatalysts, and parasitic light absorption. In the present work, carbon nanotubes (CNTs) were shown to function as an effective solid-state electron mediator in a Z-scheme OWS system. Based on the high stability and good charge transfer characteristics of CNTs, this system exhibited superior OWS performance compared with other systems using more common electron mediators. The as-constructed system evolved stoichiometric amounts of H_2_ and O_2_ at near-ambient pressure with a solar-to-hydrogen energy conversion efficiency of 0.15%. The OWS reaction was also promoted in the case that this CNT-based Z-scheme system was immobilized on a substrate. Hence, CNTs are a viable electron mediator material for large-scale Z-scheme OWS systems.

## Introduction

Overall water splitting (OWS) using solar energy and photocatalysts has attracted great interest because this process is a potential means of producing H_2_ fuel in a sustainable manner.^1−3^ Two-step excitation systems, also known as Z-scheme systems, are considered to represent a promising approach to photocatalytic OWS.^4−6^ Because the band positions of the H_2_ evolution photocatalyst (HEP) and O_2_ evolution photocatalyst (OEP) only need to meet the thermodynamic requirements for the corresponding half-reactions, a greater number of visible-light-responsive photocatalysts can be utilized in Z-scheme systems.^7^ In addition, physical separation of the H_2_ and O_2_ evolution reactions can allow cocatalysts to be tailored for each reaction, thus improving charge extraction and minimizing reverse reactions.^8^

The electron mediator is another important factor that affects the performance of Z-scheme systems. Ideally, this component should efficiently transfer photogenerated electrons and holes between the HEP and OEP. The electron mediator itself should be stable and must not induce unwanted side reactions.^9^ In earlier studies of Z-scheme systems, ionic couples were commonly used as electron mediators. Although significant OWS activity was often achieved, the use of ionic couples has some drawbacks, such as corrosion of the photocatalysts, decomposition of the ionic couples, and reverse reactions on the HEP and the OEP. Solid-state electron mediators were subsequently developed to address these concerns. Beginning in 2011, reduced graphene oxide (RGO) was used as a solid-state electron mediator for Z-scheme OWS.^10^ In later work, Au was deposited as an electron mediator for use with SrTiO_3_:La,Rh/Au/BiVO_4_:Mo sheets in Z-scheme water splitting systems.^11^ This concept provided remarkable solar-to-hydrogen energy conversion efficiency (STH) and also permitted the photocatalysts to be easily recovered after use. Because Au is expensive and tends to promote reverse reactions, indium tin oxide has also been applied as an electron mediator, although the availability of indium can be problematic.^12^ Other more easily obtainable carbon-based materials have also been utilized as solid-state electron mediators.^13^ In particular, carbon nanotubes (CNTs) have potential applications as mediators for Z-scheme systems. These materials are highly stable and exhibit excellent conductivity, which can promote efficient charge transport.^14,15^ The ease of handling of CNTs is also advantageous with regard to scale-up of the Z-scheme system. In this study, CNTs were employed as the solid-state electron mediator to construct a Z-scheme system using CoO_*x*_-loaded BiVO_4_ (Co/BVO)^16^ as the OEP and a recently developed catalyst comprising Cr_2_O_3_/Pt with IrO_2_-loaded Sm_2_Ti_2_O_5_S_2_ (Cr/Pt/STOS/Ir) as the HEP.^17^ Owing to the enhanced charge transfer efficiency obtained from this approach, the OWS activity of the as-constructed Z-scheme system was 2 orders of magnitude higher than that of a system without an electron mediator. It was also found that the CNT solvent greatly affected the performance of the Z-scheme system.

## Results and Discussion

### Characterization of CNTs

**Figure 1:**
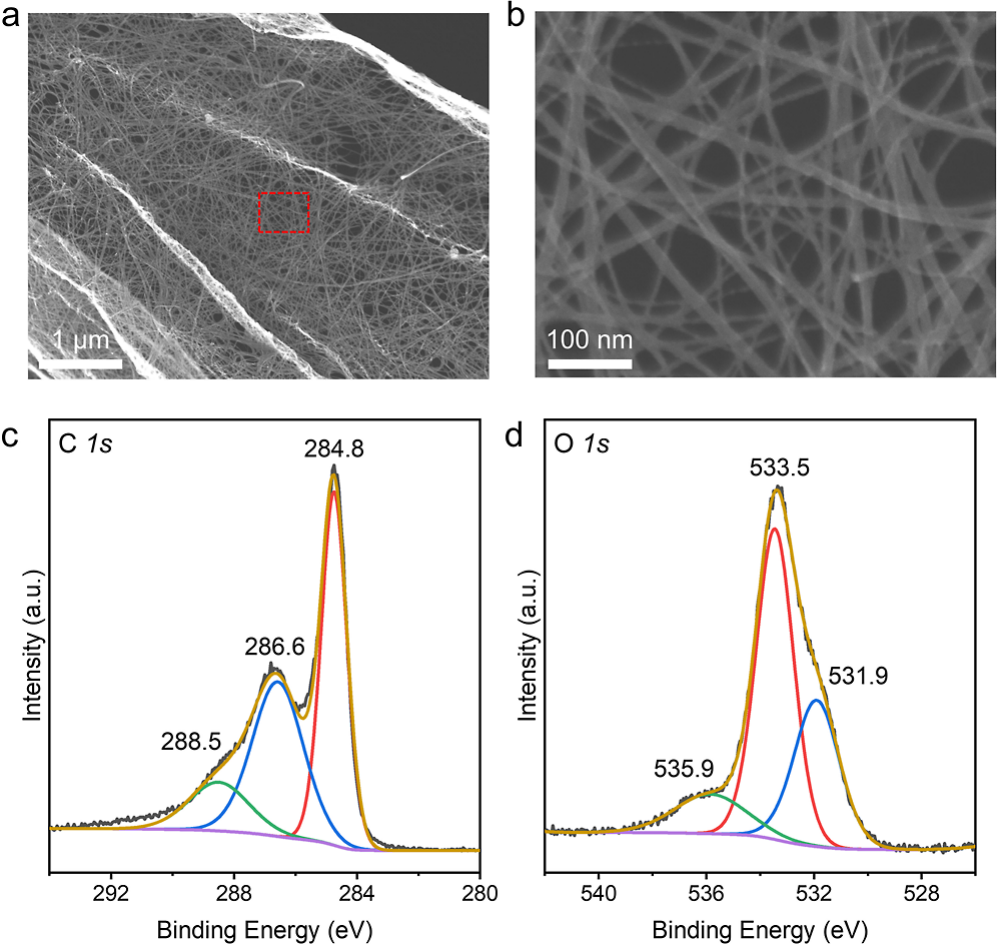
Characterization of CNTs. (a) SEM image of CNTs. (b) Enlarged view of the area in the red box in (a). (c) C 1s and (d) O 1s XPS data.

The morphologies and chemical states of the CNTs used in this work were assessed by scanning electron microscopy (SEM) and X-ray photoelectron spectroscopy (XPS). CNTs having a criss-cross morphology with diameters ranging from several to tens of nanometers were observed in the SEM images (Figure 1a,b). Closer inspection of the edges of the CNTs revealed that the larger CNTs were actually made of entanglements of several single CNTs (Figure S1). The lengths of these CNTs were in excess of several micrometers. The C 1s XPS data showed three peaks located at 284.8, 286.6, and 288.5 eV, corresponding to C–C, C–O, and C=O bonds, respectively (Figure 1c).^18^ The O 1s XPS peaks could be deconvoluted into three peaks. The main peak at 533.5 eV was ascribed to C–O bonds, while those at 531.9 and 535.9 eV originated from the C=O and H–O–H bonds, respectively (Figure 1d).^19^ The STOS used as the HEP was prepared by a flux method, and submicrometer plate-like STOS crystals were observed in SEM image (Figure S2a,b). The BVO used as the OEP was synthesized via a hydrothermal method, yielding decahedral particles with sizes on the order of 1 μm (Figure S2c,d).

### Photodeposition of CNTs on Co/BVO

**Figure 2:**
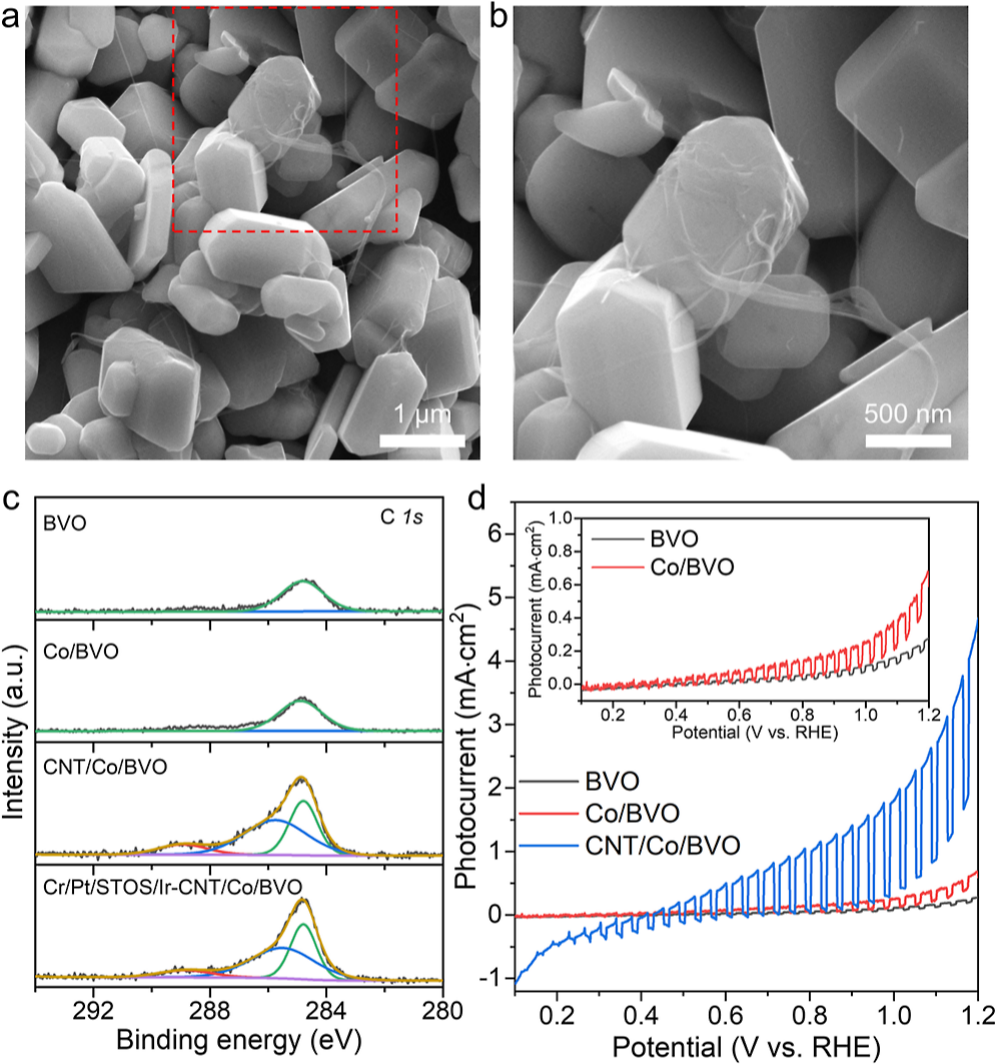
Characterization of CNT/Co/BVO. (a) SEM image of CNT/Co/BVO. (b) Enlarged view of the area in red box in (a). (c) C 1s XPS data. (d) LSV curves obtained from photoanodes under intermittent visible light irradiation. Inset: an enlarged view of the LSV curves for BVO and Co/BVO.

Prior to the construction of the Z-scheme system, a CoO_*x*_ cocatalyst was loaded onto BVO by photodeposition to promote the evolution of the O_2_. Following this, the CNTs were photoreduced on Co/BVO in an aqueous methanol solution. Subsequent to this reaction, SEM images confirmed that the CNTs were attached to the BVO surfaces (Figure 2a,b). The chemical state of carbon in CNT/Co/BVO was analyzed by XPS, using BVO and Co/BVO specimens as references. Both the BVO and Co/BVO samples generated a peak at 284.8 eV that could be assigned to adventitious carbon. In the case of CNT/Co/BVO, three XPS peaks centered at 284.8, 286.4, and 289.1 eV were obtained (Figure 2c) that were attributed to the aforementioned C–C, C–O, and C=O bonds of the CNTs, respectively. Photoelectrochemical (PEC) measurements were carried out to study the effect of the CNTs on the transfer of photogenerated electrons from Co/BVO to the Ti film acting as a back electrode. Figure 2d shows linear sweep voltammetry (LSV) curves obtained from BVO, Co/BVO, and CNT/Co/BVO photoanodes under visible light. The onset potential of the photoanodic current generated by the BVO photoanode was shifted negatively after loading the CoO_*x*_, while the photocurrent was increased by a factor of 2.3 at 1.23 V vs a reversible hydrogen electrode (RHE) (see the inset of Figure 2b). Interestingly, following the additional loading of CNTs on Co/BVO, the photocurrent was increased by a factor of 12.8 at 1.23 V vs RHE. These results confirmed that CNTs deposited on Co/BVO efficiently captured and transported electrons to the substrate.^20,21^

### Construction of the Z-Scheme System

**Figure 3:**
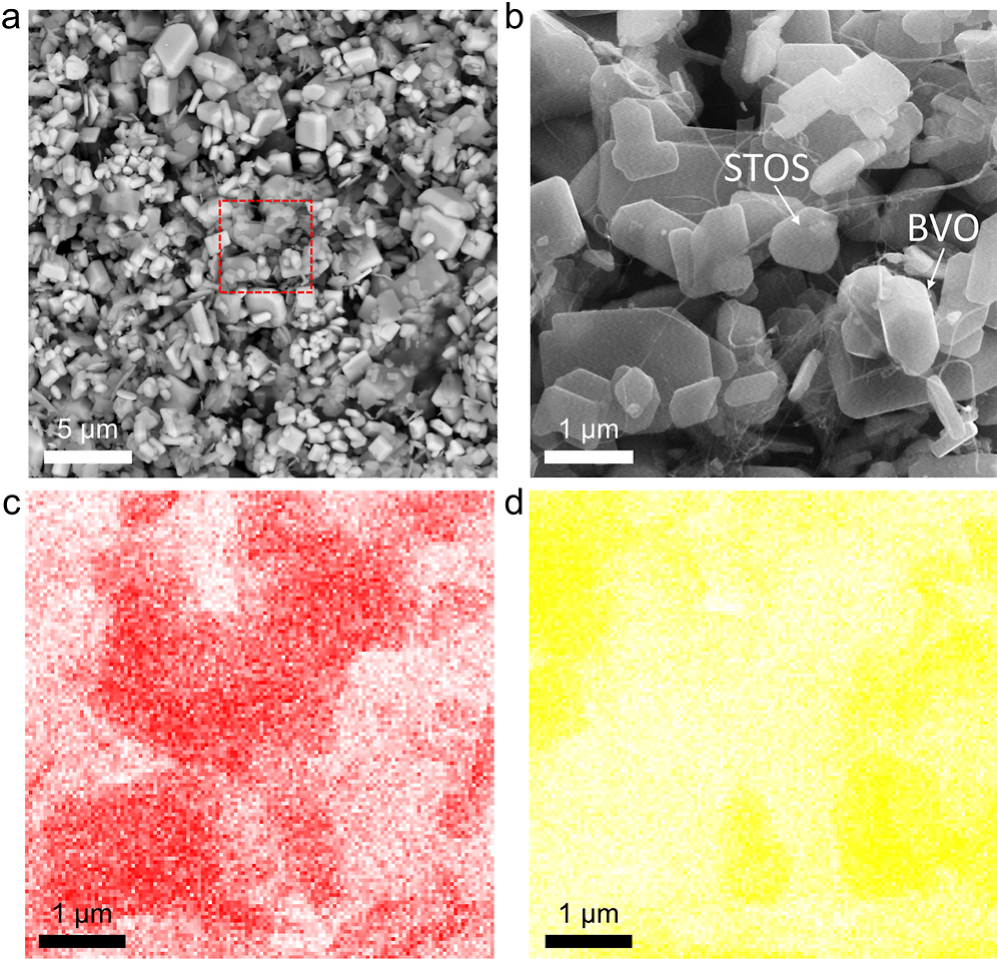
(a) Backscattering mode SEM image of the Z-scheme powder after the induction period. The bright and dark particles are the OEP and HEP, respectively. (b) Secondary electron mode SEM image showing an enlarged view of the area indicated by the red box in (a) and the corresponding EDS mapping of (c) Sm and (d) Bi.

To construct a Z-scheme system, 0.1 g of CNT/Co/BVO and 0.05 g of Cr/Pt/STOS/Ir were dispersed in 150 mL of distilled water with continuous stirring. In response to visible light, the H_2_ and O_2_ evolution rates were found to gradually increase as the reaction time was prolonged (Figure S3). This finding suggests that CNT/Co/BVO and Cr/Pt/STOS/Ir were connected by the CNTs such that charge transfer efficiency between the HEP and the OEP was enhanced during the induction period. A SEM image acquired in backscattering mode demonstrated that, after the induction period, the HEP and OEP were thoroughly mixed (Figure 3a). Analyses using the secondary electron mode clearly confirmed that the HEP and the OEP were connected by the CNTs (Figure 3b). Unlike RGO, CNTs will not excessively wrap the HEP and OEP, reducing the possibility of the blocking active surface.^17^ The elemental composition of the Z-scheme system was also confirmed by energy-dispersive X-ray spectroscopy (EDS) mapping (Figures 3c,d and S4). Figure S5 shows the UV–visible diffuse reflectance spectroscopy (DRS) data of the samples in each step during assembly of the Z-scheme system. The absorption was increased at the wavelength greater than ca. 550 nm for the physical mixture of Cr/Pt/STOS/Ir and Co/BVO mainly due to the absorption originating from the Pt cocatalyst. The absorption in this region was further increased in the case of Cr/Pt/STOS/Ir-CNT/Co/BVO due to the presence of CNTs on the Co/BVO surface. In addition, a Z-scheme-type band alignment of the HEP and the OEP was confirmed by the Mott–Schottky measurement according to our previous study (Figure S6).

### Evaluation of Photocatalytic Activity

**Figure 4:**
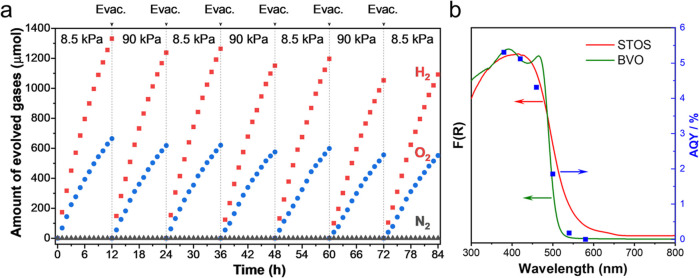
(a) Gases evolved by the Z-scheme system over time at different Ar background pressures under irradiation with >420 nm light. (b) AQY values for the Z-scheme system during OWS under Ar at a background pressure of 8.5 kPa together with DRS data for BVO and STOS.

Following the induction period, the reaction system was evacuated, and the reaction was resumed in the presence of Ar at a pressure of 8.5 kPa. H_2_ and O_2_ evolution rates of 173 and 75 μmol/h, respectively, were achieved in the first hour with the stoichiometric production of H_2_ and O_2_ after a 12 h photoreaction. At an Ar pressure of 90 kPa, 93% of the performance at 8.5 kPa was retained after 12 h. When the Ar background pressure subsequently returned to 8.5 kPa, 95% of the initial activity was still maintained (Figure 4a). Those results indicated that the background pressure had little effect on the Z-scheme water splitting system, which would be desirable with regard to practical applications. The Z-scheme system retained 82% of the initial water splitting activity after 84 h of photoreaction, demonstrating reasonable stability on the timescale of days. The apparent quantum yield (AQY) was determined to be 5.1 and 4.7% with 420 nm monochromatic light irradiation at background pressures of 8.5 and 90 kPa, respectively (Figures 4b and S7). In addition, the AQY at each wavelength agreed reasonably well with the UV–visible DRS data for BVO. This outcome implied that the performance was primarily determined by the light absorption characteristics of the OEP.^22^ BVO as the OEP has a relatively wide band gap compared with STOS used as the HEP, which limits the utilization of visible light. Exploring new OEPs with a narrow band gap is a promising way to improve the performance of the Z-scheme system. In addition, similar performances were observed among the Z-scheme systems derived from different batches of CNTs, indicating good reproducibility in the construction of the Z-scheme system (Figure S8). After the reaction, the Z-scheme powder was recovered from the aqueous solution for characterization. No significant differences in XRD or XPS results were observed before and after the reaction, further confirming the stability of the present Z-scheme system, including the cocatalysts (Figure S9 and S10). The loading amount of CNTs was further varied from 0 to 3.0 wt %. The OWS activity was gradually enhanced with increasing amount of loaded CNTs until 2.5 wt % (Figure S11). Increasing the amount further resulted in lower performance because excessive CNTs on the BVO surface would block the active sites and incident light.

The photocatalytic OWS reaction was also carried out under irradiation with a solar simulator (AM 1.5G), and STH values of 0.16 and 0.15% were obtained under background pressures of 8.5 and 90 kPa, respectively. Again, there was little impact of the background pressure on the performance of the material. After a total reaction time of 84 h, 80% of the initial activity value was maintained (Figure S12). Additionally, a CNT-based Z-scheme water splitting sheet was fabricated by immobilizing the photocatalyst powder on a glass substrate together with nanometer-sized hydrophilic silica particles. This sheet exhibited stoichiometric H_2_ and O_2_ evolution at both 8.5 and 90 kPa, although the performance of the system was inferior to those of present-day suspension systems (Figure S13). These results demonstrated that the CNT-based Z-scheme system used in this work could be easily scaled up to a large area for practical hydrogen production.^23^ The performance of the photocatalyst sheet was comparable to that of the suspension system if 20 mg of Z-scheme powder was used in each case (Figure S14). However, the gas evolution rate of the sheet was decreased when further increasing the amount of Z-scheme powder. This is most likely because the fraction of Z-scheme powder shaded by the topmost particle layer becomes higher, while the amount of Pt cocatalyst promoting the reverse reaction becomes higher with increasing particle layer thickness. Therefore, developing new fabrication routes would help to maintain the performance of the sheet with respect to the suspension system.

Additional OWS reactions were performed to investigate the factors affecting the performance of the Z-scheme system. Either Cr/Pt/STOS/Ir or Co/BVO alone did not promote the OWS (Table 1, entries 1 and 2). Without CNTs loaded on Co/BVO, the steady-state H_2_ and O_2_ evolution rates were dramatically decreased to 1.6 and 0.8 μmol/h (entry 3), respectively. These values were nearly 2 orders of magnitude lower than those obtained with the CNTs (entry 4). This effect occurred because charge transfer could only take place based on random collisions or aggregation of the HEP and OEP particles in the absence of the mediator. Besides the photodeposition method, the chemical reduction method was also applied to the loading of CNTs on Co/BVO using NaBH_4_ or H_2_ gas as the reducing agents. It was found that the performance of the Z-scheme system was dramatically decreased in both cases (Figure S15). This can be attributed to the reductive degradation of BVO by using strong reducing reagents. It is worth noting that the performance was also dramatically decreased in the case that CNTs dispersed in *N*-methyl-2-pyrrolidone (NMP) were used (entry 5). This lack of activity is attributed to poor dispersion of these CNTs in the aqueous methanol solution (Figure S16), such that well-dispersed photodeposition on Co/BVO did not occur. The loading amount of CNTs on Co/BVO was further investigated by elemental analysis. As shown in Table S1, the carbon content on Co/BVO using NMP as the solvent of CNTs stock solution was 75% of the case using water as the solvent. Moreover, it can be visually confirmed that the color of CNT/Co/BVO was more inhomogeneous when NMP was used as the solvent compared to the case when water was the solvent (Figure S17), indicating poor dispersibility of CNTs on the BVO surface. Considering that the CNTs themselves were the same, these two significant differences can explain the low performance when NMP was used as the solvent for CNTs. Therefore, the solvent used to produce the original CNT dispersion had a significant effect on the CNT photodeposition process and the subsequent performance of the Z-scheme system. These observations suggest that there is much room to promote the charge transfer ability of CNTs between the HEP and OEP by modifying the loading method and properties of the CNTs. In addition, the performance of the present Z-scheme system with CNTs as the electron mediator was higher than those reported for systems with more commonly used ionic electron mediators (entries 6 and 7). At present, the Z-scheme system based on the commercialized CNTs is comparable to that based on RGO derived from a commercial GO (G21-L, entry 8) but lower than that derived from a lab-made GO.^17^ Comparing the morphologies of the Z-scheme systems, one can come to a plausible conclusion that the connecting area between HEP and the OEP by one-dimensional CNTs is smaller than that of two-dimensional RGO sheets, resulting in the inferior charge transfer capability. However, RGO sheets tend to wrap the particles of the HEP or OEP, which can excessively cover the active surface and increase the parasitic light absorption. Such a problem can be alleviated when CNTs are used. When CNTs were photodeposited on HEP in advance under the same conditions, the H_2_ evolution rate in the Z-scheme OWS reaction of the constructed system was decreased to 61 μmol/h (Figure S18). Nevertheless, this performance was superior to the performance of Z-scheme systems without any electron mediator or constructed by physically mixing HEP, OEP, and CNTs (giving a H_2_ evolution rate of 1.6 and 8.0 μmol/h, respectively). This is because the CNTs deposited on the HEP can be partially bonded to Co/BVO after mixing and act as an electron mediator to transfer electrons from the OEP to the HEP.

**Table 1:** OWS Reaction Trials Were Performed Using Cr/Pt/STOS/Ir and Co/BVO with and without an Electron Mediator

entry	HEP^a^	electron mediator	OEP^b^	H_2_^c^ (μmol)	O_2_^c^ (μmol)
1	used			0.9	0
2			used	0	0
3	used		used	1.6	0.8
4	used	CNT-1^d^	used	175	75
5	used	CNT-2^e^	used	7.1	0
6	used	IO_3_^–^/I^–^	used	2.7	0
7	used	Fe^3+^/Fe^2+^^f^	used	114.7	54.6
8	used	RGO^g^	used	169	74

a Cr/Pt/STOS/Ir.

b Co/BVO.

c The amount of gas produced in the first hour.

d Dispersion in water.

e Dispersed in NMP.

f Adjusted to pH 2.3 by H_2_SO_4_.

g Based on commercialized GO (G21-L).^17^

### Reverse Reaction of the Z-Scheme System

By introducing stoichiometric H_2_ and O_2_ into the reaction system, the amount of H_2_ and O_2_ was maintained with a prolonged period in the presence of CNT/Co/BVO under dark conditions, indicating that CNT/Co/BVO does not promote the reverse reaction (Figure S19). Therefore, Pt on Cr/Pt/STOS/Ir is responsible for the reverse reaction according to our previous studies. Notably, the coating of Cr_2_O_3_ on Pt suppressed the reverse reaction to some extent. Therefore, developing new modification methods to more effectively inhibit the reverse reaction is an important way to improve the performance of the Z-scheme system.

### Charge Dynamics in the Z-Scheme System

**Figure 5:**
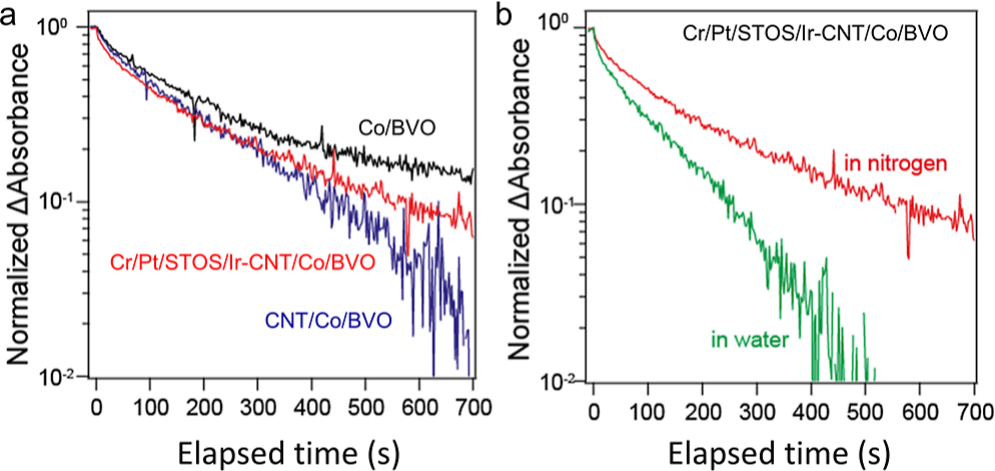
Carrier decay data for samples exposed to (a) N_2_ and (b) water vapor.

Photoinduced charge carriers in the Cr/Pt/STOS/Ir-CNT/Co/BVO system were also monitored to further assess charge transfer between the OEP and HEP following the incorporation of CNTs.^24^ As shown in Figure 5a, the decay of accumulated electrons in Co/BVO was slow as a result of the physical separation of electrons (in the BVO) and holes (in the CoO_*x*_). Following the loading of CNTs on Co/BVO, the decay of electrons was noticeably accelerated. These results indicated that the CNTs were able to capture electrons in BVO, leading to a decrease in the electron population in this material. Consequently, the separation of electrons and holes in the CNT/Co/BVO system was improved. It should be noted that in the HEP-CNTs-OEP Z-scheme system excited electrons originated from both the HEP and the OEP, as demonstrated in Figure S20. The decay of these electrons was affected by the Z-scheme recombination process, meaning that electrons in Co/BVO recombined with holes in the STOS via the CNTs. The decay of accumulated electrons in the present Z-scheme system was evidently slowed (Figure 5a), indicating that recombination proceeded in this system using CNTs as a mediator.^25^ In addition, the accumulated electrons decayed more rapidly in the case in which the HEP-CNTs-OEP sample was exposed to water vapor (Figure 5b). This phenomenon was ascribed to electron-consuming reactions induced by water (i.e., water reduction) stemming from the effectiveness of the CNTs in promoting Z-scheme recombination. These results suggest that this system should be suitable as an approach to water splitting. For comparison, the Z-scheme system was also assembled by the physical mixture of Cr/Pt/STOS/Ir, Co/BVO, and CNTs. In this case, the photoreduction of CNTs by BVO becomes difficult due to the lack of a sacrificial electron donor. Therefore, the HEP, OEP, and CNTs are randomly distributed in water, and the charge transfer can only proceed by agglomeration or collision, resulting in the poor charge transfer efficiency. In addition, no induction period was found in a reaction using the physical mixture of the three components, further confirming the poor binding of CNTs to the OEP or HEP (Figure S21a). By photoinduced absorption spectroscopy, it is found that the decay of accumulated electrons in the Z-scheme system with CNTs loaded by physical mixing was faster than the case of CNTs loaded by photodeposition (Figure S21b). Since the signals mainly originate from the electrons remaining in STOS, those results further indicate that the Z-scheme recombination (between electrons in the OEP and holes in the HEP) is ineffective, leading to the accelerated recombination of electrons and holes within STOS, which is in good agreement with the low OWS performance obtained for this Z-scheme system. The transient absorption spectroscopy analysis also showed a consistent tendency that the population of photoexcited electrons in the Z-scheme system was also significantly lower when CNTs were loaded by the physical mixing method than when CNTs were loaded by the photodeposition method (Figure S21c).

## Conclusions

In conclusion, CNTs were used as solid-state electron mediators in a Z-scheme OWS system. This system continued to exhibit an OWS activity at nearly ambient pressure and also demonstrated reasonable stability. The CNTs efficiently captured and transferred electrons from Co/BVO to Cr/Pt/STOS/Ir, increasing the activity by 2 orders of magnitude. This Z-scheme system was also able to split water into H_2_ and O_2_ when immobilized in the form of a scalable sheet. Notably, in our preliminary study, it was found that CNTs also served as electron mediators for Z-scheme systems using other oxysulfides or (oxy)nitrides. These advantages suggest applications for CNTs as efficient electron mediators in Z-scheme water splitting under practical operating conditions.

## Supporting Information Available

Experimental details; additional characterization data; and performance results (PDF)
