# Supplementary material for: Carbon Nanotubes as a Solid-State Electron Mediator for Visible-Light-Driven Z-Scheme Overall Water Splitting
Source: J Am Chem Soc. 2024 May 15;146(21):14829–34. doi: 10.1021/jacs.4c03437 (PMC11141554; doi:10.1021/jacs.4c03437)
Supplement: Supplementary file 1 — ja4c03437_si_001.pdf [file ja4c03437_si_001.pdf]

Carbon Nanotubes as a Solid-State Electron Mediator for Visible-Light-Driven Z-Scheme Overall Water Splitting

Lihua Lin[a], Yiwen Ma[a], Nobuyuki Zettsu[b,c], Junie Jhon M. Vequizo[a], Chen Gu[a], Akira Yamakata[d], Takashi Hisatomi[a], Tsuyoshi Takata[a], and Kazunari Domen[a,e]*

[a]Research Initiative for Supra-Materials, Interdisciplinary Cluster for Cutting Edge Research, Shinshu University, Nagano 380-8553, Japan

[b]Department of Materials Chemistry, Faculty of Engineering, Shinshu University, Nagano 380-8553, Japan;

[c]Energy Land-scape Architectonics Brain Bank, Shinshu University, Nagano 380-8553, Japan

[d]Faculty of Natural Science and Technology, Okayama University, Kita-ku, Okayama 700-8530, Japan

[e]Office of University Professors, The University of Tokyo, Tokyo 113-8656, Japan

* Email: domen@shinshu-u.ac.jp

Experimental Details

1. Preparation of carbon nanotubes

The carbon nanotube (CNT) sample used in the present study was provided by the Meijo Nano Carbon Co., Ltd. This material was prepared by the enhanced direction injection pyrolytic synthesis method.[1] Briefly, in this process, ferrocene was used as the catalyst with thiophene as the feedstock and toluene as the solvent. The feedstock was directly injected into a vertical tube reactor via a spray-nozzle using H_2 as the carrier gas. The reaction temperature was maintained at 1200 °C while the flange at the top of the reactor was cooled with water to below 100 °C. The product was carried out of the hot zone of the reactor by the carrier gas and collected at the bottom of the reactor, which was equipped with a stainless-steel mesh filter. The resulting CNTs were subsequently dispersed in H_2O or N-methyl-2-pyrrolidone (NMP) at a concentration of 0.2 wt% in preparation for use. These dispersions are referred to herein as CNT-1 (product no. EC-PB-0.2%) and CNT-2 (product no. EC-PBN-0.2%), respectively. Unless noted otherwise, the CNT-1 dispersion was used in the present Z-scheme system.

2. Preparation of $BiVO_4$ by a hydrothermal method and surface modification with a Co cocatalyst and CNTs

$BiVO_4$ (BVO) was prepared by dissolving NH_4VO_3 (10 mmol) and $Bi(NO_3)_3 \cdot 5H_2O$ (10 mmol) in 2.0 M aqueous HNO_3 after which the pH of the solution was adjusted to approximately 0.5 with an aqueous ammonia solution (28-30 wt%). A pale-yellow precipitate was formed after stirring for 2 h, which was followed by hydrothermal treatment at 200 °C for 24 h in a Teflon-lined stainless steel autoclave. After being allowed to naturally cool to room temperature, the product was washed with distilled (DI) water several times and then dried at 40 °C under vacuum.

Cobalt species were loaded on the BVO as a co-catalyst via a photodeposition method. In this process, a quantity of BVO powder (0.2 g) was dispersed in 150 mL of a potassium phosphate buffer solution (PBS) (pH 6.0, 50 mM) containing $Co(NO_3)_2 \cdot 6H_2O$ as the cobalt source, at a concentration of 3.0 wt% Co with respect to

the amount of BVO. The resulting dispersion was irradiated with a 300 W Xe lamp with the full-arc for 1 h and then washed and filtered to obtain the Co/BVO.

A portion of the Co/BVO (0.1 g) was subsequently dispersed in an aqueous methanol solution (50 vol%) containing nominal 0.5, 1.0, 2.5 or 3.0 wt% CNTs with respect to the mass of Co/BVO. Unless noted otherwise, 2.5 wt% CNTs was added to the aqueous methanol solution. This dispersion was then irradiated using a 300 W Xe lamp with the full-arc for 3 h, after which the CNT/Co/BVO powder was separated from the solution by centrifugation.

When CNTs were loaded on BVO by chemical reduction method, $NaBH_4$ and H_2 gas were used as reducing agents. In the former case, 2.5 wt% CNTs were impregnated on the BVO surface. The obtained powder was dispersed in 30 mL of an aqueous $NaBH_4$ solution (2.0 $mg{\cdot}mL^{-1}$). After stirring for 30 min, the solution was filtered and the obtained powder was dried under vacuum at 40°C for 12 h. In the latter case, 2.5 wt% CNTs were impregnated on the BVO surface and then the obtained powder was heated at 400 ^{o}C for 1 h under the flowing gas mixture of H_2 (20 $mL{\cdot}min^{-1}$) and N_2 (100 $mL{\cdot}min^{-1}$).

3. Preparation of $Sm_2Ti_2O_5S_2$ by a flux method and surface modification

The $Sm_2Ti_2O_5S_2$ (STOS) powder used in this work was synthesized by grinding a mixture of Sm_2O_3 (0.489 g, Wako), Sm_2S_3 (0.215 g, High Purity Chemicals) and TiO_2 (0.295 g, Rare Metallic Co., Ltd.), corresponding to a molar ratio of 1:2:6, in a glove box filled with N_2. Following this, sulfur powder (0.1 g, High Purity Chemicals), corresponding to 10 wt% with respect to the mass of the $Y_2Ti_2O_5S_2$ product, was added to obtain a sulfur-rich environment. This precursor mixture was then combined with a $LiCl/CaCl_2$ flux (42.4:57.6 mass ratio, Wako) at a precursor-to-flux mass ratio of 1:3. The mixture was then sealed under vacuum in an evacuated quartz tube and calcined at 700 °C for 24 h. Subsequently, the sintered samples were washed with DI water and filtered to obtain the STOS powder and then dried in a vacuum oven. Following this,

the STOS was calcined in air at 200 °C followed by washing with 47% H_2SO_4 to remove excess sulfur.

Following the above, a portion of the STOS powder (0.2 g) was dispersed in 15 mL of H_2O in a glass vial. A set amount of an aqueous $IrCl_3$ solution (sufficient to give 0.5 wt% Ir with respect to the mass of STOS) was added and the glass vial was heated at 150 °C for 15 min in a microwave reactor (Monowave 400, Anton Paar). The powdered product was subsequently recovered by filtration. The resulting STOS/Ir was dispersed in a glass vial filled with 15 mL of ethylene glycol (EG), after which a specific amount of an aqueous $H_2PtCl_6 \cdot 6H_2O$ solution (sufficient to give 1.0 wt% Pt with respect to the mass of STOS) was added. The vial was then heated at 150 °C for 15 min in the same microwave reactor. Following this, the Pt/STOS/Ir was collected by filtration. Finally, the Pt/STOS/Ir (0.05 g) was dispersed in 150 mL of a 10 vol% aqueous methanol solution under full-arc irradiation by a 300 W xenon lamp using K_2CrO_4 as a precursor (equivalent to 0.5 wt% Cr with respect to the mass of STOS). After a 1 h photoreaction, the sample was collected by filtration and washed several times with DI water.

4. Construction of a Z-scheme system for overall water splitting

Quantities of the CNT/Co/BVO (0.1 g) and Cr/Pt/STOS/Ir (0.05 g) were added to 150 mL of DI water and irradiated using a 300 W Xe lamp with a cut-off filter (L42, $\lambda \geq$ 420 nm) for 12 h to promote the connection of the HEP and OEP via the CNTs. The material obtained from drying the product is referred to herein as the Z-scheme powder. For physical mixing method, the Co/BVO (0.1 g) and Cr/Pt/STOS/Ir (0.05 g) together with 2.5 wt% CNTs with respect to the amount of Co/BVO were added to 150 mL of DI water and irradiated using a 300 W Xe lamp with a cut-off filter (L42, $\lambda \geq$ 420 nm) for 12 h. The IO_3^-/I^- and Fe^{3+}/Fe^{2+} ionic couples were produced by dispersing the Co/BVO (0.1 g) and Cr/Pt/STOS/Ir (0.05 g) in 150 mL of 2.5 mM NaI or 2.0 mM $FeCl_3$ aqueous solutions, respectively. The pH was not adjusted in trials with the I_3^-/I^- couple but was adjusted to 2.3 with H_2SO_4 when working with the Fe^{3+}/Fe^{2+} couple.

5. Preparation of photocatalyst sheets

A quantity of the Z-scheme powder (20 mg) and nanometer-sized silica particles (40 mg) were dispersed in 1 mL of DI water and ultrasonicated for 5 min. The resulting suspension was then dropped onto a glass substrate and subsequently dried on a hot plate at 50 °C.

6. Photocatalytic reactions during Z-scheme overall water splitting

Water splitting reactions were performed in a Pyrex top-illuminated reaction vessel connected to a closed gas circulation system. In these trials, a portion of the Z-scheme powder (0.15 g) was dispersed in 150 mL of DI water without any pH adjustment and the dispersion was maintained at room temperature by circulating cooling water. After evacuating air from the system, the suspension was irradiated by a 300 W xenon lamp equipped with a cut-off filter (L42, $\lambda \geq 420$ nm). The total background pressure of water vapor and Ar gas was adjusted to approximately 8.5 or 90 kPa in different trials. The gas phase products were analyzed using an integrated gas chromatography system consisting of a GC-8A chromatograph (Shimadzu Corp.) equipped with molecular sieve 5A columns and a thermal conductivity detector, with argon as the carrier gas. In some experiments, the reaction vessel was illuminated with AM 1.5G simulated sunlight. The top window of the reaction cell was covered with aluminum foil to limit the illuminated sample area to 9 cm^2.

Similarly, in experiments with the photocatalyst sheet, the sheet was placed at the bottom of the reaction cell, which was filled with 40 mL of DI water. The temperature of the system was maintained at room temperature by circulating cooling water. After evacuating air from the system, the total background pressure of water vapor and Ar gas was adjusted to 8.5 or 90 kPa. The system was illuminated by a 300 W xenon lamp equipped with a cut-off filter (L42, $\lambda \geq 420$ nm). For comparison, 20 mg of the Z-scheme powder was dispersed in 40 mL of DI water in a small reactor with an irradiation area of 9 cm^2 to set up a suspension system with similar reaction conditions to the evaluation of the sheet system.

7. Apparent quantum yield measurements

The apparent quantum yield (AQY) associated with Z-scheme water splitting by the two-step photoexcitation system was calculated as

$$\text{AQY (\%)} = [A\times R]/I\times 100, \qquad (1)$$

where R and I represent the gas evolution rate and incident photon flux, respectively. In the case of the H_2-producing half-reaction, the coefficient A is the number of electrons consumed to produce a hydrogen molecule (that is, 2) while the value for the O_2-producing half-reaction is the number of holes consumed to produce an oxygen molecule (4). For Z-scheme overall water splitting, the coefficient A is 4 for H_2 evolution. The total intensity of irradiation was measured using a grating spectroradiometer (LS-100, Eko Instruments). The AQY for overall water splitting was determined using 300 W xenon lamp as the light source but with various bandpass filters. The irradiation area was maintained at 9 cm^2.

8. Solar-to-hydrogen energy conversion efficiency measurements

The water splitting reaction trials were carried out under simulated sunlight (AM 1.5G). The solar-to-hydrogen energy conversion efficiency (STH) values were calculated as

$$\text{STH (\%)} = (R(H_2)\times\Delta G_r)/(P\times S)\times 100 \ (2),$$

where $R(H_2)$, ΔG_r, P and S represent the hydrogen evolution rate during the overall water splitting reaction, the Gibbs energy change for the reaction $H_2O(l) \rightarrow H_2(g) + 1/2\ O_2(g)$, the energy intensity of the AM 1.5G solar irradiation (100 mW/cm^2) and the irradiation area (9 cm^2), respectively. The ΔG_r value used for the calculations was 237 kJ mol^{-1}.

9. Photoelectrochemical assessments

BVO, Co/BVO and CNT/Co/BVO photoanodes were prepared using a particle transfer method. In this process, a Ti layer was deposited by vacuum evaporation on a powder layer that had been drop-cast onto a glass substrate. The powder layer was then transferred to another glass plate using adhesive aluminum tape. Finally, the

unnecessary part of the electrode was covered with epoxy resin. A three-electrode setup connected to a potentiostat (HSV-110, Hokuto Denko) was employed to carry out linear sweep voltammetry (LSV) trials with the prepared photoanodes, with Pt wire and a Ag/AgCl electrode as the counter and reference electrodes, respectively. An aqueous 0.5 M K_2SO_4 solution buffered with potassium phosphate at a pH of 7 was used as the electrolyte. A 300 W Xe lamp (CERMAX PE300BF, PerkinElmer) with a cutoff filter (HOYA) was used as the light source. Mott-Schottky plot was used to determine the band position of STOS. The STOS electrode was prepared by the particle transfer method with Ti substrate and the measurement was performed in 0.1 mol·L^{-1} Na_2SO_4 aqueous solution on BioLogic VSP-300 electrochemical system.

10. Characterization

Powder XRD analyses were carried out with a Rigaku MiniFlex 300. The morphology of the samples was studied using FE-SEM (Hitachi SU8000 and Phenom Pharos, ThermoFisher Scientific). EDS mapping was recorded using Phenom Pharos. XPS analyses were carried out with a PHI Quantera II instrument (ULVAC-PHI, Inc.) employing a monochromatized Al Kα line source. UV-visible DRS data were acquired using a JASCO V-670 UV-visible system. Elemental analysis of carbon was carried out using an EMIA-Pro apparatus (Horiba).

11. Transient absorption spectroscopy

The photoinduced charge carriers in the excited samples were studied using a custom-built Fourier transform infrared (IR) system equipped with a beam splitter, moving mirror and LED lamp for excitation.[2] The accumulated electrons in the samples were probed at 802 cm^{-1} (approximately 0.1 eV). During these experiments, the samples were irradiated at 470 nm by a continuous wave light emitting diode (CW-LED). Photocarriers were generated by irradiating each sample continuously for 223 s using this device at a power density of 40 mW/cm^2, following which irradiation was stopped to allow the decay kinetics of the accumulated electrons to be monitored. The aperture size of the IR

beam used for probing was 4 mm and the IR radiation from the photoexcited sample was detected by a mercury cadmium telluride device. The time resolution of the spectrometer was set to 2.23 s. This film specimens for analysis were prepared by homogeneously dispersing a portion of the photocatalyst powder in water with subsequent drop-casting of this suspension onto a CaF_2 substrate. The resulting specimen was then dried in air overnight to obtain a powder film having a density of 0.995 mg/cm^2. The as-prepared film on the CaF_2 substrate was subsequently placed in a stainless-steel reaction cell that was evacuated before admitting N_2 gas or H_2O vapor to a cell pressure of 10 Torr prior to the measurements. The data were acquired at room temperature. As for the decay profiles of photoexcited electrons probed at 5000 nm in Z-scheme systems with different loading methods of CNTs, the samples were excited by 470 nm laser pulses (fluence: 0.16 mJ/pulse, ns-Surelite laser system, repetition: 1 s). The measurement was carried out at room temperature and under N_2 atmosphere (pressure: 20 Torr).

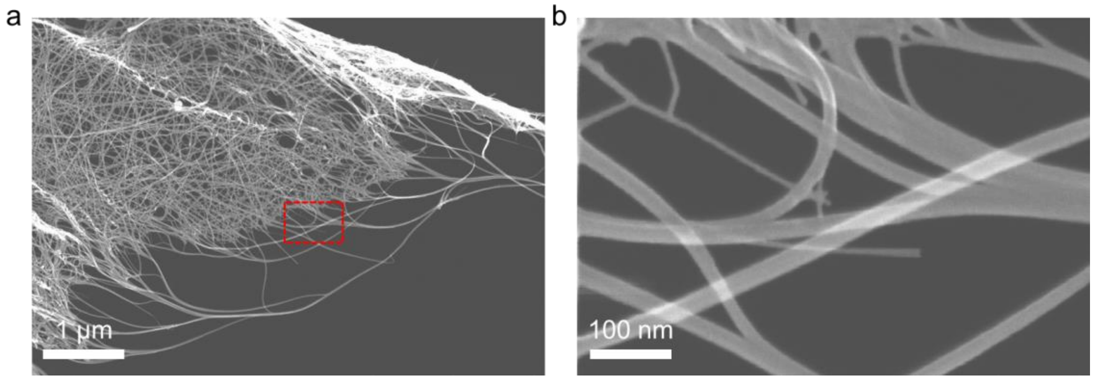

Figure S1. (a) SEM image of CNTs used in this work. (b) Enlarged view of area within red box.

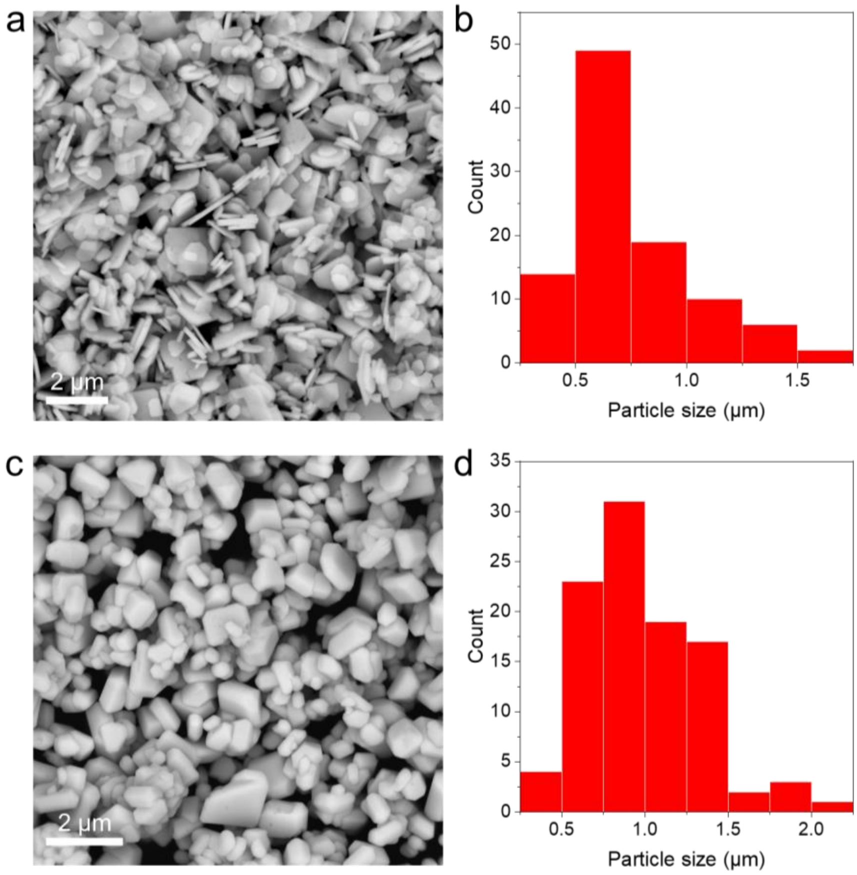

Figure S2. (a) SEM image of a STOS sample and (b) long-axis particle size distribution for STOS sample based on 100 particles. (c) SEM image of BVO sample and (d) long-axis particle size distribution for BVO sample based on 100 particles.

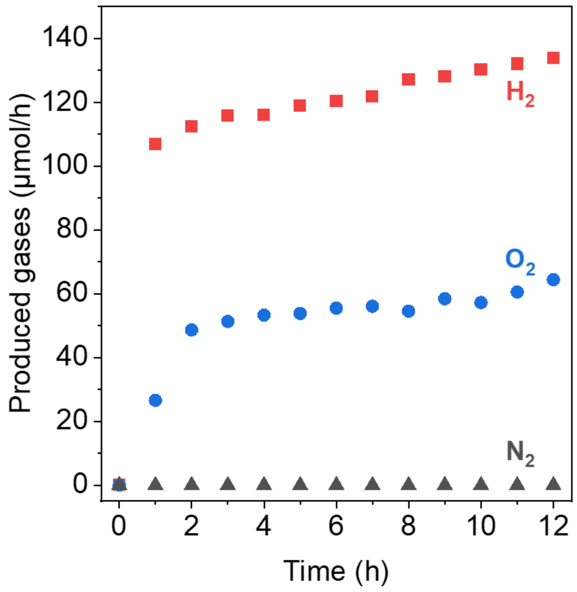

Figure S3. Gas evolution rates for Z-scheme system over time during induction period with exposure to >420 nm light.

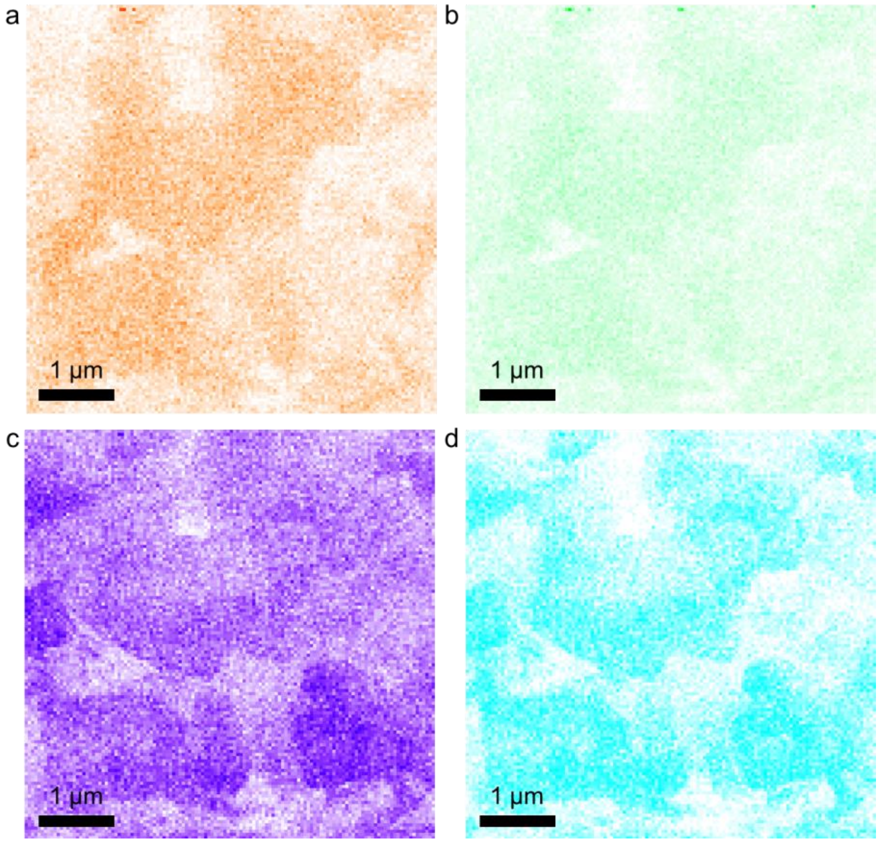

Figure S4. (a) Ti, (b) S, (c) V, and (d) O EDS maps for Z-scheme catalyst.

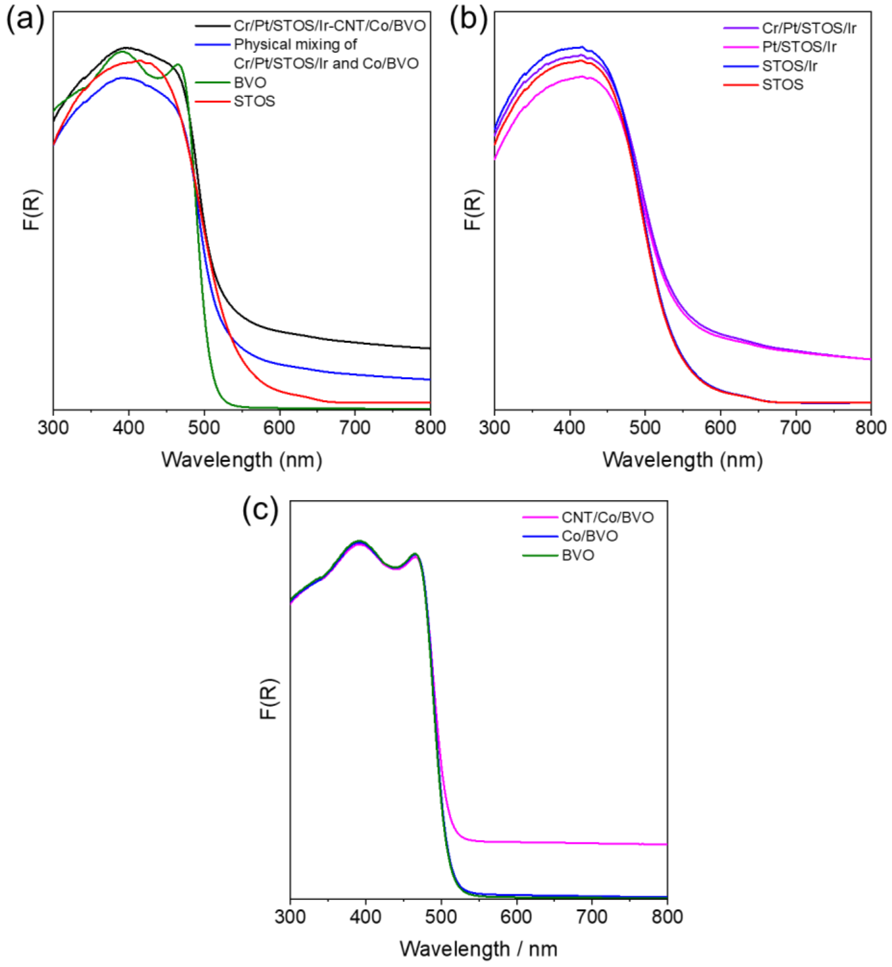

Figure S5. UV-visible DRS data of (a) Z-scheme system without and with photodeposition of CNTs on Co/BVO, (b) STOS and (c) BVO without and with surface modifications.

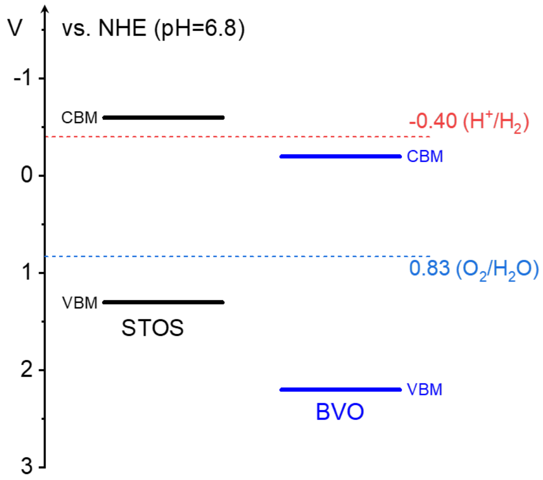

Figure S6. The band alignment of the Z-scheme system.

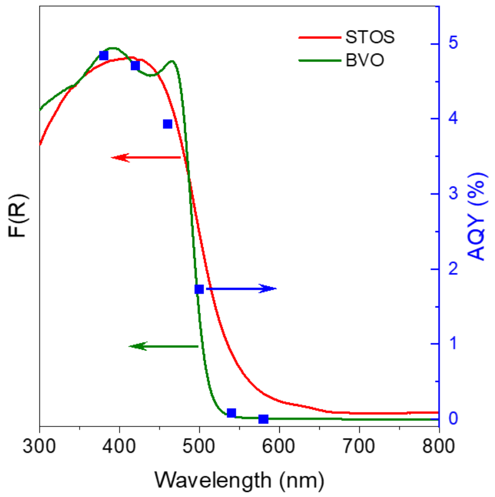

Figure S7. AQY values for Z-scheme system at Ar background pressure of 90 kPa together with DRS data for BVO and STOS.

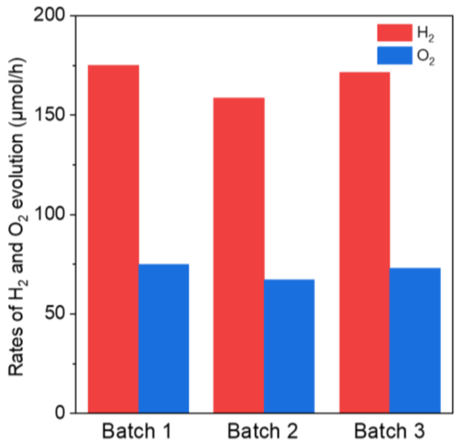

Figure S8. Z-scheme OWS performance with different batches of CNTs.

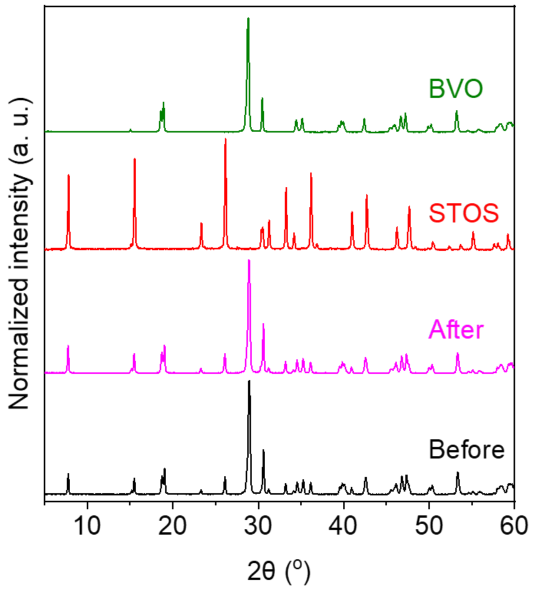

Figure S9. XRD patterns for Z-scheme catalyst before and after an 84 h photoreaction together with patterns for STOS and BVO as references.

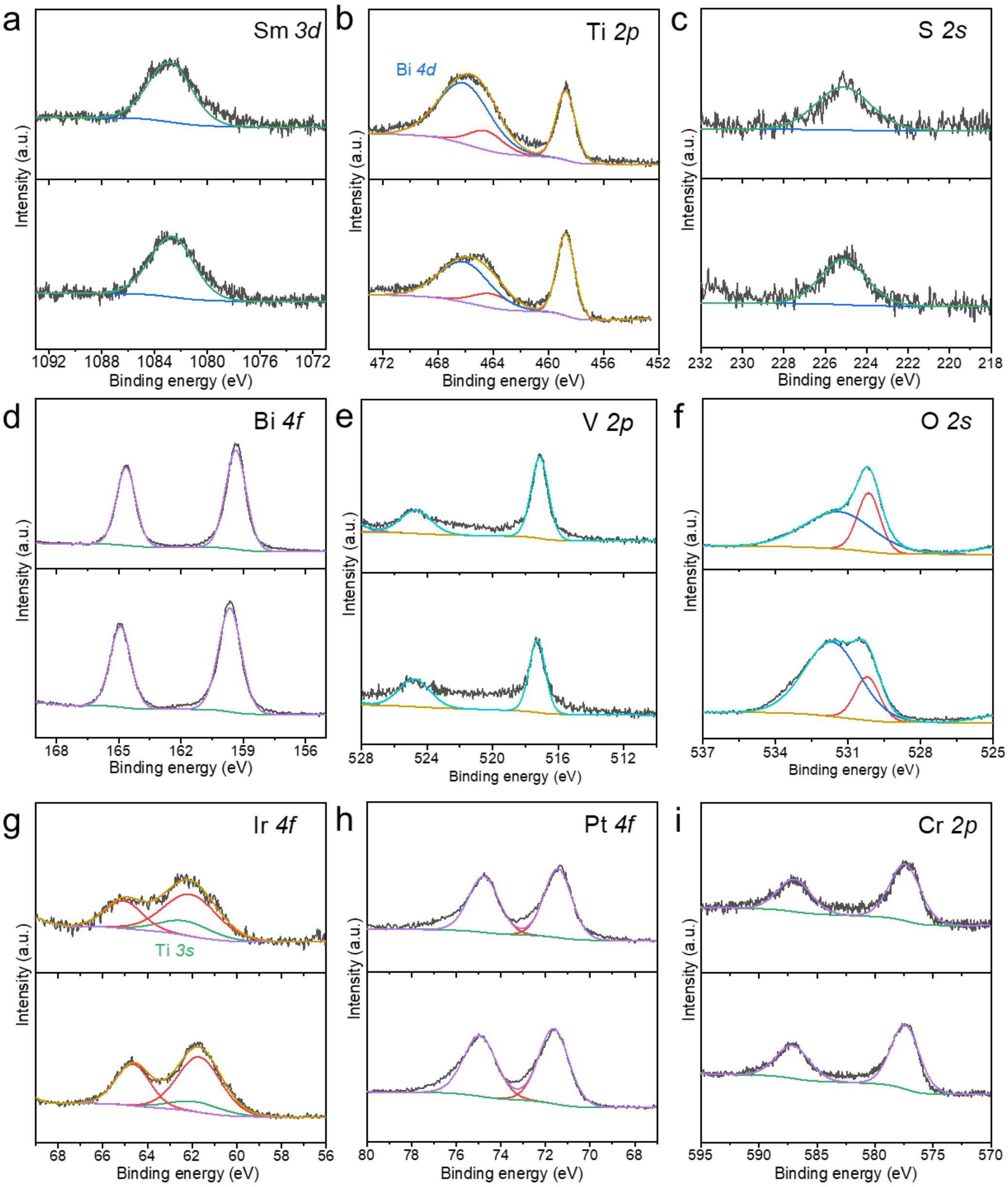

Figure S10. (a) Sm 3*d*, (b) Ti 2*p*, (c) S 2*s*, (d) Bi 4*f*, I V 2*p*, (f) O 1*s*, (g) Ir 4*f*, (h) Pt 4*f*, and (i) Cr 2*p* XPS peaks before (upper) and after (lower) 84 h photoreaction.

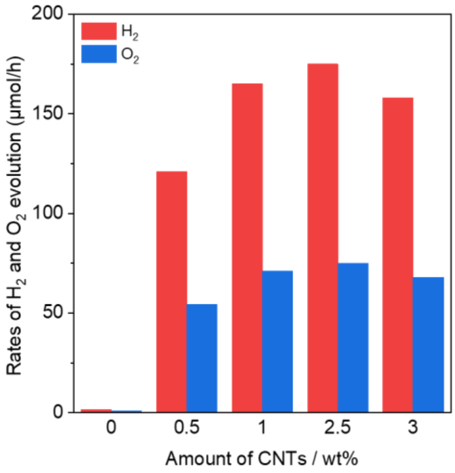

Figure S11. The dependence of Z-scheme OWS performance on the quantity of CNTs added.

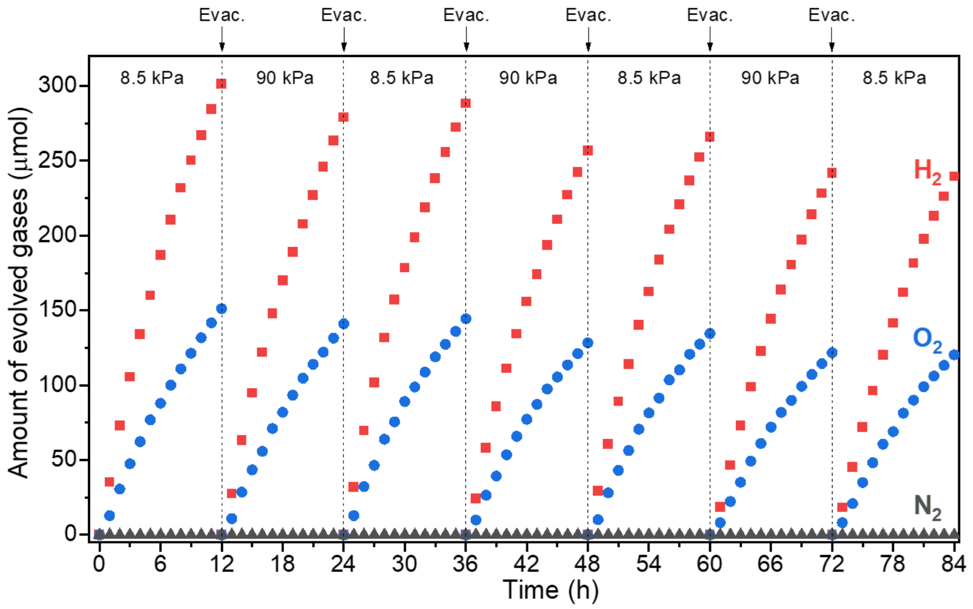

Figure S12. Gases evolved by Z-scheme system over time at different Ar background pressures with irradiation by AM1.5 solar simulator.

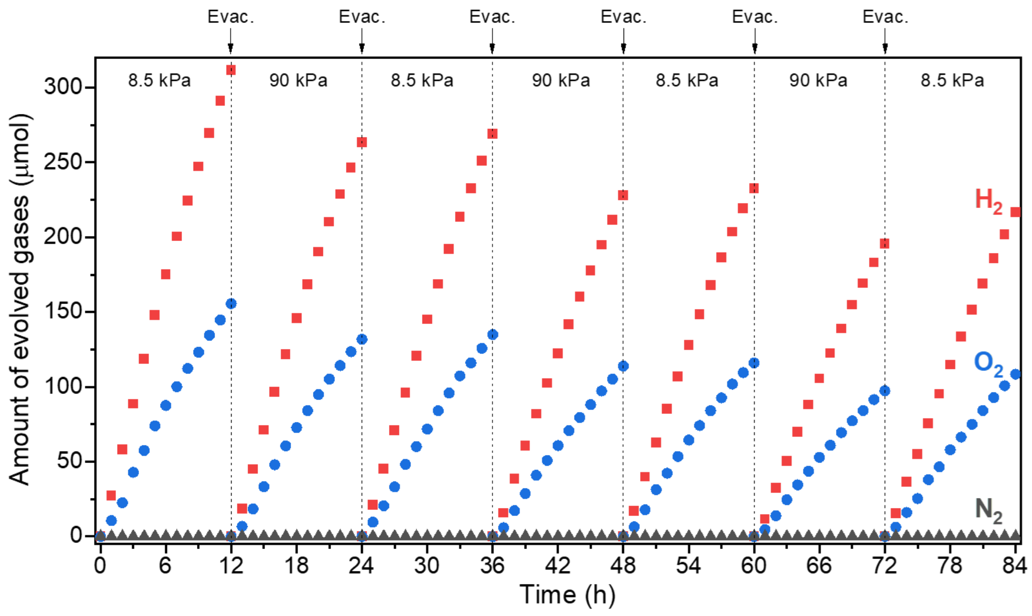

Figure S13. Gases evolved by Z-scheme photocatalyst sheet over time at different Ar background pressures with exposure to >420 nm light.

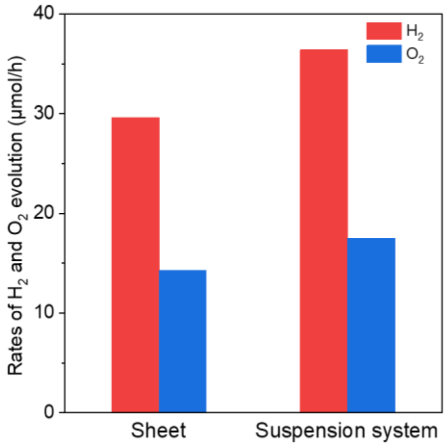

Figure S14. Z-scheme OWS performance of photocatalyst sheet and suspension system consisting of 20 mg of Z-scheme powder under similar reaction conditions.

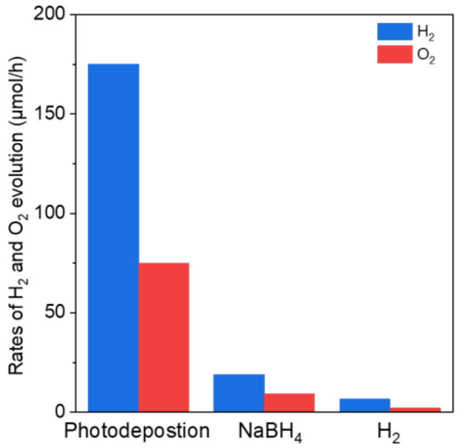

Figure S15. OWS activity of the Z-scheme system with Co/BVO loaded with CNTs by different reduction methods.

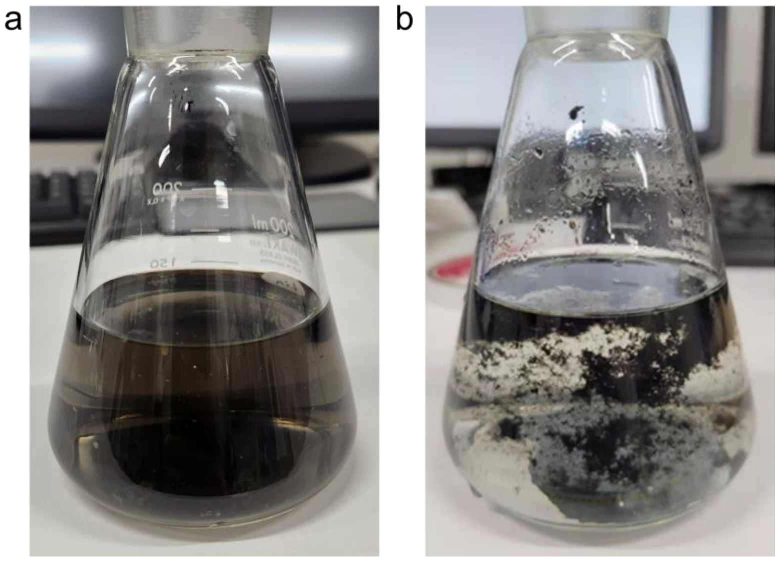

Figure S16. Photographic images of (a) CNT-1 and (b) CNT-2 dispersions in aqueous methanol solutions.

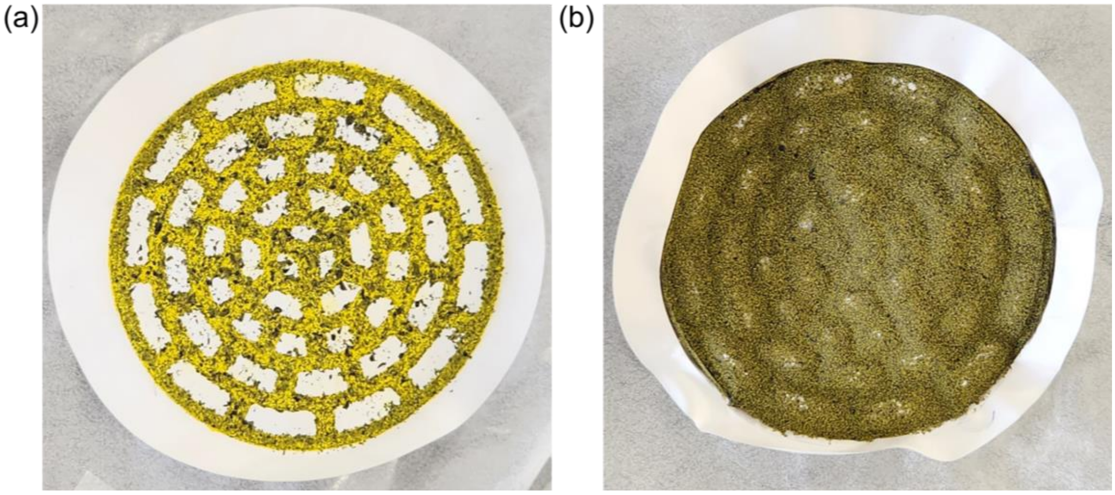

Figure S17. Photographs of CNT/Co/BVO when using (a) NMP and (b) water as the solvent of the CNTs stock solution.

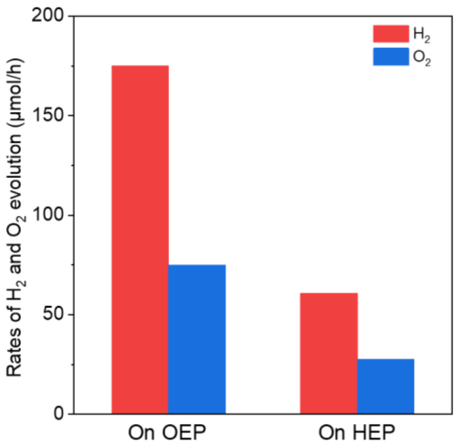

Figure S18. OWS activity of the Z-scheme system with CNTs loaded on OEP or HEP by a photodeposition method.

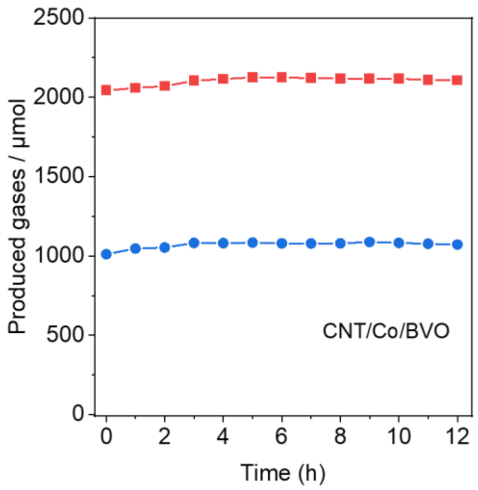

Figure S19. Dark reaction of CNT/Co/BVO by introducing around 2 mmol H_2 and 1 mmol O_2 in the reaction system.

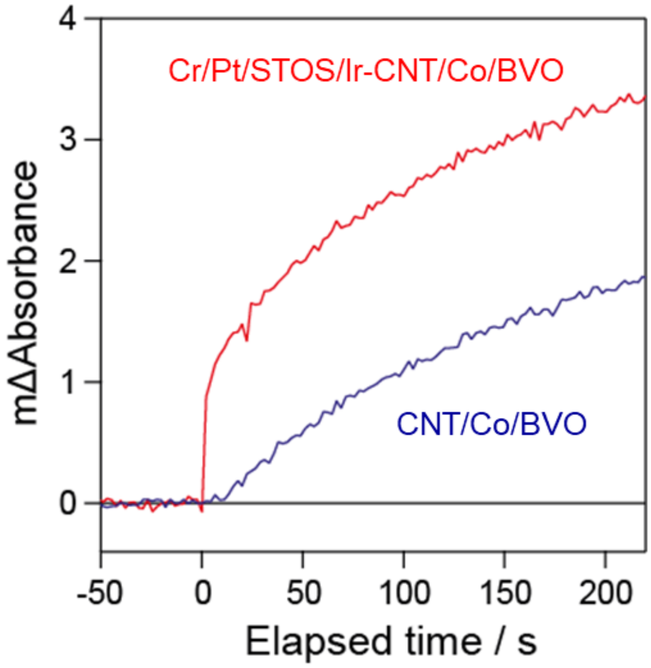

Figure S20. Data showing photoinduced absorption of accumulated electrons in CNT/Co/BVO and Cr/Pt/STOS/Ir-CNT/Co/BVO specimens. The samples were excited using a 470 nm CW-LED for 223 s and electrons were probed at 802 cm^{-1} (approximately 0.1 eV). Data were collected at room temperature and under N_2 at a pressure of 10 Torr.

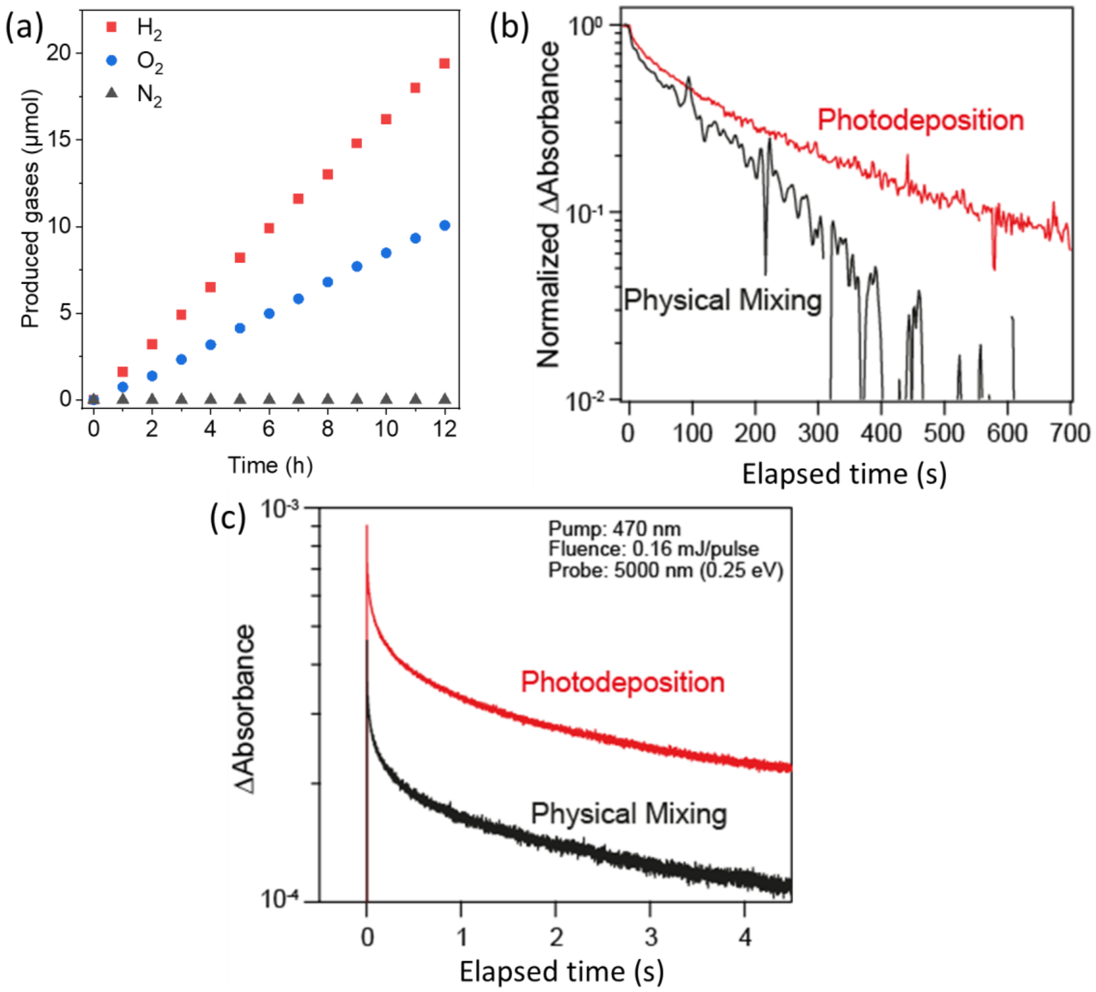

Figure S21. (a) Gases evolved in initial 12 h from Z-scheme system constructed by physical mixing of HEP, OEP and CNTs. (b) Photoinduced absorption of accumulated electrons in Z-scheme systems with different loading methods of CNTs. The samples were excited by 470 nm CW-LED for 223 s. Electrons were probed at 802 cm^{-1} (~0.1 eV). The measurement was carried out at room temperature and under N2 atmosphere (pressure: 10 Torr). (c) Decay profiles of photoexcited electrons probed at 5000 nm in Z-scheme systems with different loading methods of CNTs. The samples were excited by 470 nm laser pulses (fluence: 0.16 mJ/pulse, ns-Surelite laser system, repetition: 1 s). The measurement was carried out at room temperature and under N_2 atmosphere (pressure: 20 Torr).

Table S1. The amounts of nominal loaded CNTs and carbon content on Co/BVO.

Sample	Solvent of CNTs stock solution	Nominal loaded amount of CNTs (%)	Carbon content (%)
CNT/Co/BVO	Water	2.5	2.0
CNT/Co/BVO	NMP	2.5	1.5

Reference

(1) Saito, T.; Ohshima, S.; Okazaki, T.; Ohmori, S.; Yumura, M.; Iijima, S. Selective Diameter Control of Single-Walled Carbon Nanotubes in the Gas-Phase Synthesis. *J. Nanosci. Nanotechnol.* **2008**, *8*, 6153-6157

(2) Lin, L.; Ma, Y.; Vequizo, J. J. M.; Nakabayashi, M.; Gu, C.; Tao, X.; Yoshida, H.; Pihosh, Y.; Nishina, Y.; Yamakata, A.; et al. Efficient and stable visible-light-driven Z-scheme overall water splitting using an oxysulfide H_2 evolution photocatalyst. *Nat. Commun.* **2024**, *15*, 397
